# High-dimensional deconstruction of pancreatic cancer identifies tumor microenvironmental and developmental stemness features that predict survival

**DOI:** 10.1038/s41698-023-00455-z

**Published:** 2023-10-19

**Authors:** Erik P. Storrs, Prathamesh Chati, Abul Usmani, Ian Sloan, Bradley A. Krasnick, Ramandeep Babbra, Peter K. Harris, Chloe M. Sachs, Faridi Qaium, Deyali Chatterjee, Chris Wetzel, S. Peter Goedegebuure, Thomas Hollander, Hephzibah Anthony, Jennifer Ponce, Ateeq M. Khaliq, Shahed Badiyan, Hyun Kim, David G. Denardo, Gabriel D. Lang, Natalie D. Cosgrove, Vladimir M. Kushnir, Dayna S. Early, Ashiq Masood, Kian-Huat Lim, William G. Hawkins, Li Ding, Ryan C. Fields, Koushik K. Das, Aadel A. Chaudhuri

**Affiliations:** 1 Department of Genetics, Washington University School of Medicine, St. Louis, MO USA; 2 Division of Biology and Biomedical Sciences, Washington University School of Medicine, St. Louis, MO USA; 3 Department of Radiation Oncology, Washington University School of Medicine, St. Louis, MO USA; 4 Division of Gastroenterology, Department of Medicine, Washington University School of Medicine, St. Louis, MO USA; 5 Department of Surgery, Washington University School of Medicine, St. Louis, MO USA; 6 Division of Hematology & Oncology, Department of Medicine, University of Rochester Medical Center, Rochester, NY USA; 7 Division of Laboratory Medicine, Department of Pathology, MD Anderson Cancer Center, Houston, TX USA; 8 Siteman Cancer Center, Washington University School of Medicine, St. Louis, MO USA; 9 McDonnell Genome Institute, Washington University School of Medicine, St. Louis, MO USA; 10 Division of Hematology/Oncology, Department of Medicine, Indiana University School of Medicine, Indianapolis, IN USA; 11 Department of Pathology and Immunology, Washington University School of Medicine, St. Louis, MO USA; 12 Division of Oncology, Department of Medicine, Washington University School of Medicine, St. Louis, MO USA; 13 Department of Biomedical Engineering, Washington University in St. Louis, St. Louis, MO USA; 14 Department of Computer Science and Engineering, Washington University in St. Louis, St. Louis, MO USA

**Keywords:** Cancer microenvironment, Pancreatic cancer

## Abstract

Numerous cell states are known to comprise the pancreatic ductal adenocarcinoma (PDAC) tumor microenvironment (TME). However, the developmental stemness and co-occurrence of these cell states remain poorly defined. Here, we performed single-cell RNA sequencing (scRNA-seq) on a cohort of treatment-naive PDAC time-of-diagnosis endoscopic ultrasound-guided fine needle biopsy (EUS-FNB) samples (*n* = 25). We then combined these samples with surgical resection (*n* = 6) and publicly available samples to increase statistical power (*n* = 80). Following annotation into 25 distinct cell states, cells were scored for developmental stemness, and a customized version of the Ecotyper tool was used to identify communities of co-occurring cell states in bulk RNA-seq samples (*n* = 268). We discovered a tumor microenvironmental community comprised of aggressive basal-like malignant cells, tumor-promoting SPP1+ macrophages, and myofibroblastic cancer-associated fibroblasts associated with especially poor prognosis. We also found a developmental stemness continuum with implications for survival that is present in both malignant cells and cancer-associated fibroblasts (CAFs). We further demonstrated that high-dimensional analyses predictive of survival are feasible using standard-of-care, time-of-diagnosis EUS-FNB specimens. In summary, we identified tumor microenvironmental and developmental stemness characteristics from a high-dimensional gene expression analysis of PDAC using human tissue specimens, including time-of-diagnosis EUS-FNB samples. These reveal new connections between tumor microenvironmental composition, CAF and malignant cell stemness, and patient survival that could lead to better upfront risk stratification and more personalized upfront clinical decision-making.

## Introduction

Pancreatic ductal adenocarcinoma (PDAC) is the third leading cause of cancer death in the United States, with a 5-year survival rate of 10.8%^1^. PDAC has remained largely refractory to available therapeutics, with a hallmark of heterogeneous chemotherapeutic responses in subsets of patients^2^. Over the past decade, bulk tumor sequencing has enabled annotation of the genomic landscape in PDAC^3,4^. This has led to several classification systems for PDAC^3,5,6^. The general consensus consistently demonstrates the existence of two major subtypes of PDAC: the classical or pancreatic progenitor subtype associated with a relatively better prognosis (characterized by differentiated ductal markers like PDX1) and the basal-like, squamous, or quasi-mesenchymal subtype associated with a poorer prognosis (characterized by the expression of basal-like markers like cytokeratin 81 (KRT81))^3,4^. While these insights have allowed for the elucidation of unique transcriptional networks^7,8^, they have yet to allow for the development of effective clinical interventions^9^. Underlying this, in part, is the fact that these subtyping techniques rely on gross analysis of bulk sequencing data, creating blind spots in individual cell states and features of individual cells within a tumor sample. This issue is especially pronounced in PDAC, where only 20% of a sample may be tumor cells, and thus the ability to fully decipher all cellular variants is limited when using traditional next-generation sequencing (NGS) methodologies^4^.

Advances in single-cell RNA sequencing (scRNA-seq) have provided the ability to describe individual cell profiles and query individual cell states^10–13^, enabling a more in-depth analysis of the tumor microenvironment (TME) and tumor heterogeneity. Several efforts have demonstrated that PDAC tumors are a heterogeneous and spatially diverse admixture of “basal-like” and “classical” cells with the potential for plasticity between transcriptomic states with unknown prognostic implications^14–16^. Recent studies also show that a variety of cell states have implications for TME to tumor cell interactions, such as cancer-associated fibroblasts (CAFs)^17–20^ and tumor-associated macrophages (TAMs)^14^. These cell states have also been described in terms of developmental status, with more basal-like malignant cells displaying EMT-like characteristics and CAFs segregating into more and less plastic stroma^3,21,22^.

In this work, we identify TME communities and developmental stemness characteristics in PDAC that reveal new connections between tumor microenvironmental composition, CAF, and malignant cell stemness and predict patient survival.

## Results

### Cellular makeup of the PDAC TME

**Fig. 1:**
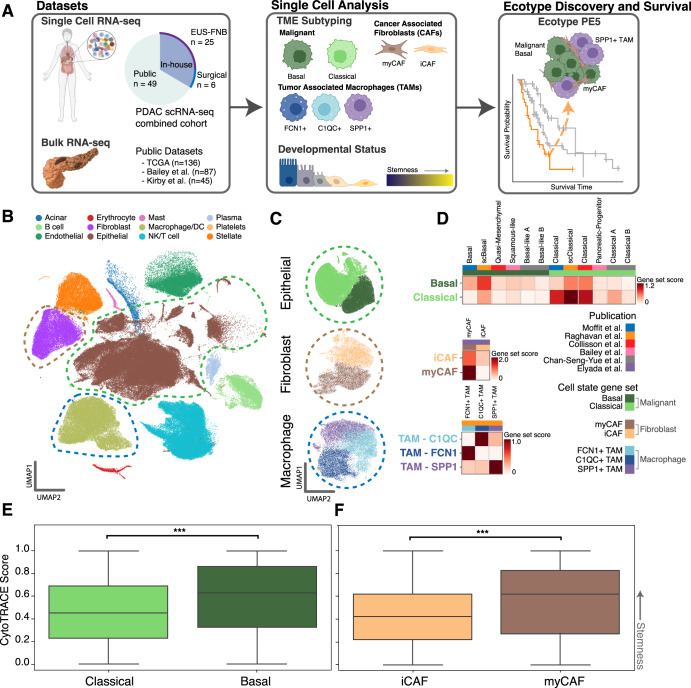
Study overview and pancreatic cancer single-cell analysis. **A** Single-cell RNA sequencing (scRNA-seq) was performed on treatment-naïve pancreatic ductal adenocarcinoma (PDAC) tumor tissue samples acquired by esophageal ultrasound-guided fine needle biopsy (*n* = 25). These were integrated with in-house surgical resection PDAC samples from six patients and samples from three publicly available PDAC scRNA-seq datasets resulting in a combined dataset of ~190k cells from 80 independent PDAC patients. The resulting data were used to identify PDAC cell states, including malignant and immune subtypes based on gene sets and known expression markers from published studies. With single-cell annotations in hand, we determined fibroblast and malignant cell states and stemness and used a modified version of the Ecotyper tool to identify co-occurring patterns of cell states (termed ecotypes) in bulk RNA-seq samples. We found that pancreatic ecotype PE5, comprised of Malignant Basal-like cells, myCAFs, and SPP1+ TAMs, was associated with worse survival. **B** UMAP decomposition of scRNA-seq expression profiles. **C** Regenerated and sub-clustered UMAP plots for malignant, cancer-associated fibroblast (CAF), and tumor-associated macrophage (TAM) cell states. **D** Gene set scores from published data for the previously mentioned cell states. **E**, **F** CytoTRACE developmental stemness scores for CAF and malignant cell states. Higher values indicate more stem-like cells. *** indicates *p*-value << 0.005 as calculated by the Wilcoxon rank sum test. The upper and lower bounds signify the first and third quartiles, respectively. The median is denoted by the center line. The whiskers represent data points within 1.5 times the interquartile range.

We performed scRNA-seq of PDAC from standard-of-care time-of-diagnosis endoscopic ultrasound-guided fine needle biopsy (EUS-FNB) specimens at the time of diagnosis and from surgical samples obtained from tumor resections to enable a clinically integrated, comprehensive view of PDAC (Fig. 1A, Supplementary Table 1). In total, we acquired 31,215 cells across 25 independent PDAC patients for our in-house EUS-FNB cohort and 11,353 cells from 6 independent PDAC patients for our in-house surgical cohort. All samples were acquired from primary tumors. To increase power, we then combined the in-house scRNA-seq data with three publicly available datasets: Peng et al., Chan-Seng-Yue et al., and Lin et al.^11–13^ (Supplementary Table 2), increasing our sample size to a total of 198k cells from 80 independent PDAC tumors. The integrated dataset was clustered and annotated using known cell type markers, resulting in the labeling of 12 cell types. (Fig. 1B). These clusters showed representation from samples across datasets, indicating that dataset-specific batch effects were largely removed during integration (Supplementary Fig. 1). Normal epithelial cells were identified via CNA alteration content with CopyKAT^23^ and normal epithelial markers^20^ and were excluded from downstream analyses (Supplementary Fig. 2A).

The Malignant, NK/T cell, macrophage/DC, and fibroblast clusters were further sub-clustered into more granular cell states. Cell states were identified based on a combination of known marker genes and gene set scores from the literature (Fig. 1C, D, Supplementary Table 4). For malignant cells, we partitioned cells into classical and basal-like subtypes based on gene expression similarity with previously published bulk subtypes^3,5,6^. Fibroblasts were split into myofibroblast (myCAF) and inflammatory (iCAF) fibroblast populations based on gene sets from Elyada et al.; we did not see the expression of MHC-II genes indicative of antigen-presenting fibroblasts (apCAFs)^17^. Macrophages/DCs were split into the following states based on marker genes and gene set scores from Raghaven et al.^14^: TAM–C1QC+, TAM–FCN1+, TAM–SPP1+, TAM–proliferating, DC, and pDC. The NK/T cell cluster was subdivided into CD4, CD8, CD8 exhausted, CD4/CD8 proliferating, Treg, and NK cell states based on the presence of known marker genes.

### Stemness in malignant and fibroblast cell states

We then determined the developmental stemness of the malignant and CAF subclusters. We used CytoTRACE^24^, a computational tool, to obtain a developmental stemness score for each cell, with cells having a high CytoTRACE score being more stem-like and those with a low CytoTRACE score being less stem-like. We found that basal-like malignant cells were more stem-like than their classical counterparts (Fig. 1E, Supplementary Table 5). This difference has been previously suggested in the literature, with the more aggressive basal-like subtype being more likely to undergo epithelial–mesenchymal transition (EMT), resulting in higher rates of metastasis^25,26^. Interestingly, we also found that myCAFs are significantly more stem-like than iCAFs (Fig. 1F).

To investigate this developmental stemness continuum further, we performed pathway analysis on DEGs between iCAF and myCAF populations. Notably, pathways involved in ECM organization, cell differentiation, and EMT transition were upregulated in myCAFs (Supplementary Fig. 3B, C). The presence of these pathways suggests that myCAFs retain a more mesenchymal stem-like phenotype than the more developmentally mature iCAFs. To further interrogate genes contributing to CAF differentiation, we identified the genes most highly correlated with CytoTRACE score (Supplementary Table 6). We found that expression of *ACTG1*, *TMSB10*, *S100A11*, and *ACTB* are highly correlated with CytoTRACE developmental score in CAFs and are involved in cell motility, adhesion, and proliferation. Additionally, *TMSB10* is known to promote M2 macrophage conversion in lung adenocarcinoma^27^. Furthermore, the genes *ENO1* and *LGALS1* correlated strongly with developmental stemness and have been previously described by Grünwald et al. as markers associated with CAF plasticity^21^. Overall, these data suggest that stem-like myCAFs have a proclivity toward increased TME remodeling capacity.

### Cell state compositions associated with patient survival

**Fig. 2:**
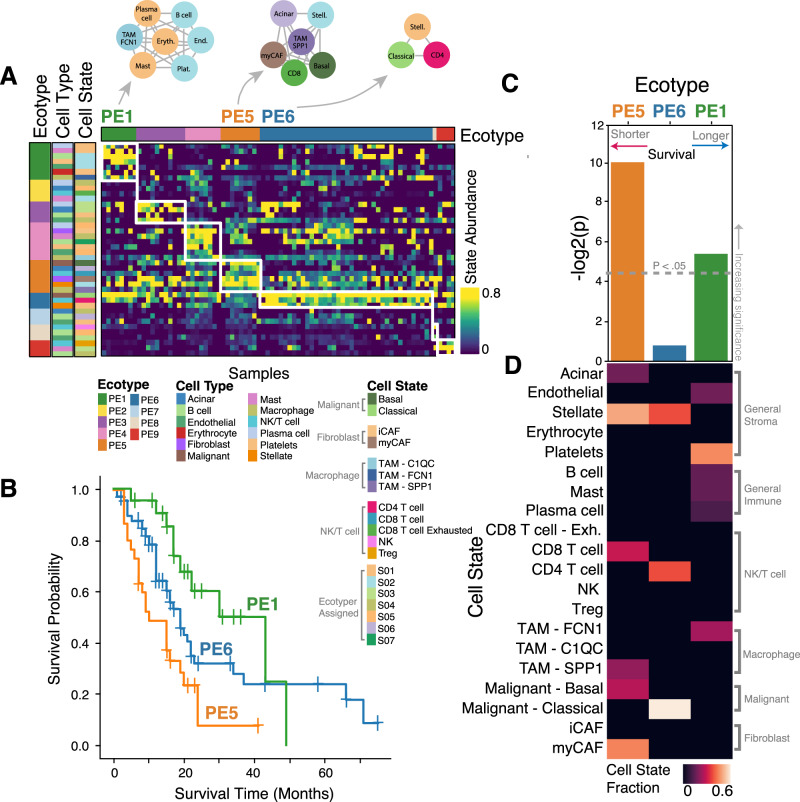
Pancreatic cancer ecotype discovery and survival analysis. **A** Ecotypes discovered within our pancreatic cancer single-cell RNA-seq dataset (*n* = 190 K cells) and their association with cell state abundances. **B** Kaplan–Meier curves showing patient survival in PDAC patients profiled by TCGA, stratified by the dominant ecotype (PE1, PE5, or PE6) measured in surgical tumor resection tissue. **C** −log2 (*p*-value) associated with overall survival for each of these pancreatic ecotypes in TCGA. **D** Fraction of each cell state within each pancreatic ecotype in scRNA-seq expression data.

Next, we extended our single-cell expression profiles to publicly available bulk expression datasets with associated clinical metadata (Supplementary Table 3) to find cell state patterns associated with patient survival. To this end, we modified the in-silico TME dissection tool EcoTyper^28^. In the published Ecotyper tool, cell states must be discovered de novo, meaning expression profiles of specific cell states cannot be defined upfront. Since this would prevent our ability to find associations of specific cell states defined in our single-cell data, we made adjustments to the Ecotyper methodology (further described in “Methods”) to allow for the specification of exactly predefined cell states. Applying our modified version of Ecotyper to our single-cell expression profiles, we grouped significantly co-occurring cell states into communities or “ecotypes” (Fig. 2A, Supplementary Tables 7–9). We thus discovered 9 distinct pancreatic ecotypes, labeled PE1-PE9, each with its own distinct pattern of cell state enrichment. Three of these ecotypes—PE1, PE5, and PE6—were also present in significant numbers of tumor tissue bulk RNA-seq samples from PDAC patients (Supplementary Fig. 4A, Supplementary Fig. 5A, B).

Notably, samples with a PE5-dominant ecotype showed consistently poor survival across all bulk RNA-seq datasets (Fig. 2B, C, Supplementary Figs. 4C, D, 6A), including at the time of diagnosis in the EUS-FNB cohort (Supplementary Fig. 4B). PE5 showed enrichment for several cell states known to be associated with aggressive tumor behavior and poor prognosis^3,29,30^, including basal-like tumor cells, myCAFs, and SPP1+ tumor-associated macrophages (Fig. 2D). PE5 also overlapped most strongly with the pan-carcinoma ecotypes shown recently by Luca et al.^28^ to be associated with worst survival (Supplementary Fig. 5D). In contrast, PE1 and PE6 were associated with better survival outcomes than PE5 (Fig. 2B, C, Supplementary Fig. 4B–D). PE1 is immune-enriched, containing plasma cells, mast cells, B cells, and FCN1+ TAMs, while PE6 contains CD4 T cells and classical malignant cells. Overall, the makeup of PE1 and PE6 corroborates known biology, with immune fraction being associated with increased survival^31^ and the relatively improved prognosis of the classical PDAC subtype compared to basal-like^3,5,6^.

We also assessed our pancreatic ecotypes in colon adenocarcinoma (COAD) (Supplementary Table 13) and head and neck squamous cell carcinoma (HNSCC) (Supplementary Table 14) patient tumors profiled by TCGA^32,33^, and in murine pancreatic cancer gene expression data from Mueller et al.^34^. Interestingly, we observed pancreatic ecotypes to be present in these three other settings (Supplementary Fig. 7A). And while there was no significant pancreatic ecotype survival association in HNSCC, we observed the same overall survival association in COAD (*p*-value = 0.027) that we saw in PDAC (Supplementary Fig. 7B, C). This suggests that the prognostic utility of pancreatic ecotypes could generalize to cancers of the gastrointestinal system, but not necessarily outside of it (Supplementary Fig. 7B, C). Additionally, we further investigated the accuracy of pancreatic ecotypes in COAD by assigning them to samples in a scRNA-seq dataset from Lee et al.^35^. Once assigned, we compared our ecotype assignments to bulk consensus molecular subtype (CMS) classifications assigned by Lee et al. (Supplementary Fig. 7D). Overall, there was an agreement between ecotype classification and CMS subtype: the aggressive PE5 ecotype overlapped most with CMS4 (the most EMT-like CMS subtype), while PE1 and PE6 were most associated with CMS1 (immune-like) and CMS2 (canonical), respectively^36^.

### Impact of developmental stemness on patient survival

**Fig. 3:**
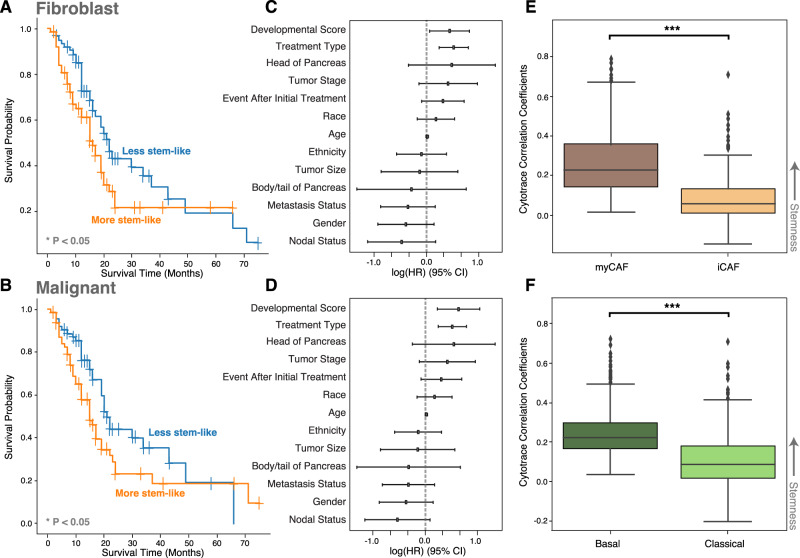
Association of CAF and malignant cell stemness with overall survival. **A**, **B** Kaplan–Meier plots for TCGA PDAC bulk RNA-seq samples when partitioned into more versus less stem-like groups of fibroblasts (*p*-value = 0.03) and malignant cells (*p*-value = 0.01). Groups were selected based on the average Cytotrace correlation of cell type-specific genes. The median score was used as a threshold to partition the two groups. **C**, **D** Multivariate Cox regression hazard ratios and confidence intervals for fibroblast (*p*-value = 0.03) and malignant (*p*-value = 0.01) developmental stemness scores in PDAC TCGA while also including clinical features. **E**, **F** Distribution of CytoTRACE stemness correlation coefficients for fibroblast and malignant cell state-specific genes identified by Ecotyper in PDAC single-cell RNA-seq data. *** indicates *p*-value << 0.005 as calculated by the Wilcoxon rank sum test. The upper and lower bounds signify the first and third quartiles, respectively. The median is denoted by the center line. The whiskers represent data points within 1.5 times the interquartile range.

Given the developmental continuum in CAF and malignant cell states found in our single-cell data, along with pancreatic ecotype PE5’s enrichment for myCAFs and basal-like malignant cells, we sought to more directly quantify the impact of developmental stemness on patient survival for these cell states. To do so, we calculated a developmental stemness score for bulk RNA-seq samples based on the expression of developmental stemness-associated genes in CAF and malignant cell states that we learned from scRNA-seq data analyzed by CytoTRACE (Fig. 1E, Supplementary Table 6). When partitioned into low vs. high differentiation groups based on this developmental stemness score, we observed inferior survival with more stem-like CAFs or more stem-like malignant cells across PDAC bulk RNA-seq cohorts (Fig. 3A–D, Supplementary Fig. 8A–D, Supplementary Table 11). Additionally, when genes specific to CAF and malignant cell states were correlated with CytoTRACE scores from the PDAC single-cell data, the states associated with PE5 (myCAF and malignant basal-like) were significantly more stem-like than their non-PE5 related cell states (Fig. 3E, F). These data highlight the importance of the developmental stemness continuum in both malignant and CAF cell states as it pertains to survival in pancreatic cancer.

## Discussion

Using microarrays, Moffitt et al. categorized PDAC into classical and basal-like populations^3^. Multiple groups have since performed bulk RNA sequencing to corroborate these findings and identify potential other tumor cell subtypes^5,6,12,14^. Unlike bulk RNA-seq, scRNA-seq allows us to individually profile each cell and thus appreciate the full breadth, granularity, and diversity in cell states and profiles within the tumor microenvironment. This is especially important in a cancer like PDAC, where only ~20% of cells in a biopsy sample are tumor cells, with the remaining cells representing various components of the TME^37^. Thus, in this study, we followed our scRNA-seq analysis of 80 patients, including 25 EUS-FNB biopsies obtained at the time of diagnosis and 6 surgical resections, with digital dissection of bulk expression profiles from 268 predominantly early-stage and surgically resected PDAC tumors.

While scRNA-seq has traditionally been felt to be impractical within the clinical workflow of patients, we have demonstrated with a collaborative, multidisciplinary approach that not only is scRNA-seq feasible but also high-dimensional deconstruction is clinically actionable from standard-of-care EUS-FNB samples at the time of diagnosis. With only 1–2 additional passes, adding less than ~5 min to the procedure time and no increased morbidity, samples can be routinely acquired when obtaining a diagnosis. Among the 25 in-house patients processed, PE1/6 and PE5 subtypes were identified and showed similar trends to the bulk cohorts, with PE5-dominant samples showing worse overall survival. This may allow for more personalized clinical decision-making for patients starting from the time of diagnosis.

By performing both scRNA-seq and bulk RNA-seq on such a large scale, we not only recapitulated known tumor subtypes (classical and basal-like) but also uncovered a spectrum of tumor heterogeneity with PDAC tumors harboring different mixtures of malignant subtypes and TME cell states. Furthermore, we discovered a developmental dichotomy in malignant and CAF cell states that we identified using CytoTRACE^24^. By modifying the Ecotyper framework^28^, we then inferred ecotypes in nearly 300 PDAC patients, where we found a pancreatic ecotype (PE5) that conferred a significantly worse prognosis as compared to other pancreatic ecotypes.

This aggressive PE5 pancreatic ecotype was enriched for malignant basal-like, myCAF, and SPP1+ TAM cell states. It is tempting to speculate that identifying tumors enriched for the PE5 ecotype could be used to personalize more aggressive targeted systemic therapy regimens and closer monitoring for tumor progression in these high-risk patients.

In contrast to PE5, PE1 and PE6 were associated with improved survival. PE1 is immune-enriched and predominantly composed of immune cell states like plasma cells, mast cells, B cells, and FCN1+ TAMs. Previous literature has shown that higher immune cell fractions and lower tumor cell fractions are associated with a positive prognosis^31^. Additionally, PE6 is significantly enriched for classical malignant cells, which are less aggressive than their basal-like counterparts in PE5^3,5,12^.

Additionally, we showed that the CAF and malignant cell states within PE5 are less differentiated and more stem cell-like, suggesting that tumors associated with inferior survival harbor an environment supportive of more EMT-like cell states. We also showed that the expression of a gene set associated with these immature cell states is indicative of worse survival for patients. Gulati et al. demonstrated that knocking down genes associated with the immature malignant cell state led to decreased tumor growth in vivo in a breast cancer xenograft model^24^. Similar methods could potentially be applied to PDAC to improve clinical outcomes for otherwise high-risk patients.

This study has several limitations. First, we utilized publicly available sequencing and clinical-correlative data in addition to performing in-house scRNA-seq of 31 tumor samples. While all public data utilized for this study were previously published and also secondarily analyzed^4–6,11,13,38^, it will be important to further corroborate our findings in a prospective setting. Second, scRNA-seq from time-of-diagnosis EUS-FNB samples is technically challenging given the limited sample material obtained. While we demonstrated promising ability to risk-stratify patients at the time of diagnosis by deconvolving pseudo-bulked scRNA-seq data, it will be important to corroborate these findings with bulk RNA-seq. Third, the biomarker-based survival data we show here are correlative in nature. It will be important to perform clinical trials in the future where, for example, patients with PE5-like tumors are selected to receive treatments targeting molecular pathways specific to TAM, myCAF, or more stem-like malignant or CAF cell states.

In summary, we identified pancreatic ecotypes and developmental continuums from a large-scale high-dimensional analysis of PDAC RNA sequencing data, including time-of-diagnosis EUS-FNB specimens, that revealed connections between tumor microenvironmental composition, malignant cell and CAF developmental stemness, and patient survival that could lead to better upfront risk stratification and more personalized clinical decision-making in the future.

## Methods

### PDAC tumor collection and processing

Following written informed consent, endoscopic ultrasound was performed on patients with suspected solid pancreatic masses based on CT or MRI imaging (Fig. 1A, Supplementary Table 1). The diagnosis of pancreatic adenocarcinoma was confirmed by a formal pathologic evaluation. After clinical diagnostic tissue acquisition was completed with 2–3 passes of a 22-gauge needle, an additional pass was obtained with a backfin “fine-needle biopsy” (FNB) needle. Tissue was carefully washed with cold PBS, collected in RPMI 1640 media (Gibco) on ice when processed fresh, or collected in freezing media (90%FBS + 10% DMSO, when processed at a later time point) and dissociated into single-cell suspension both mechanically and enzymatically as previously described^39^. Resected surgical tumor tissue was also dissociated in a similar way to obtain single-cell suspensions. Subsequently, single-cell suspensions were diluted to a final concentration of ~1000 cells/μl, and sequencing libraries were prepared using the 10× Genomics Chromium Single Cell 5’ library platform. Complementary DNA libraries were then sequenced on an Illumina NovaSeq S4 flow cell with a target of 50,000 reads/cell. The methods were performed in accordance with relevant guidelines and regulations and approved by the institutional review board at the Washington University in St. Louis School of Medicine.

### In-house scRNA-seq data processing

We aligned sequencing reads to the GRCh38 reference genome and obtained gene expression counts using 10× Cell Ranger V2-3.0.2 with default parameters^40^. FASTQ files were aligned to the GRCh38 reference genome with the STAR aligner^41^. Cell-specific unique molecular identifiers (UMIs) were then used to generate gene expression matrices.

### Integration of public scRNA-seq datasets

Filtered in-house EUS-FNB and surgical samples (*n* = 31) were integrated with three publicly available scRNA-seq datasets. These datasets include Peng et al.^11^, Lin et al.^13^, and Chan-Seng-Yue et al.^12^. Peng et al. was downloaded from the Genome Sequence Archive under project PRJCA001063, Lin et al. was downloaded from the GEO database at accession number GSE154778, and Chan-Seng-Yue et al. from the EGA under accession code EGAS00001002543 (Supplementary Table 2). Peng et al. FASTQ files were reprocessed in the same manner as the in-house data in the previous section. Clinical metadata used in survival analyses on the public bulk expression datasets can be found in Supplementary Tables 10–12.

First, cells expressing less than 200 total genes and genes that were expressed in fewer than 3 cells were filtered from the dataset. Additionally, cells with a mitochondrial DNA percentage of over 25% were filtered from the dataset. Doublets were removed from each sample using Scrublet. Scrublet’s scrub_doublets function was used individually on each sample with default parameters. Counts were then normalized by total count, log-transformed, and scaled. Principal components (PCs) were generated using the 3000 most variable genes. PCs were batch-corrected, and cells were integrated using the Harmony^42^ batch correction tool. These steps were performed with the Scanpy single-cell analysis library^43^.

### Cell state identification

Cell states were identified through multiple rounds of clustering. Initially, cells were clustered into macro-level cell types. For initial clustering, adjacent normal and metastasis samples were included. Following initial clustering, these samples were removed, so downstream analysis and reclustering were done with only primary tumor samples. Clustering was done using the Leiden algorithm^44^. For initial clustering, a resolution of 2 and 40 PCs was used. Clusters were merged and assigned to cell states based on known expression markers: fibroblast (BGN+, FAP+, SPARC+), NK/T cell (CD45+, CD3G+ and/or NKG7+), monocyte/DC (LYZ+, CD14+ and/or FCER1A+), Epithelial cell (EPCAM+, KRT18+), endothelial cell (PECAM1+), erythrocyte (HBA1+), B cell (MS4A1+), mast cell (CPA3+, KIT+), plasma cell (SDC1+, IGHG1+), acinar cell (PRSS1+, CDH5+), stellate cell (RGS5+), and platelets (ITGA2B+).

Four clusters were then further refined (as described below): epithelial cell, monocyte/DC, fibroblast, and NK/T cell. For each of these clusters, counts data were renormalized and batch-corrected for each cluster separately prior to reclustering of the cells using the same methodology as the previous paragraph. The top 10 PCs and a resolution of 1 were used for reclustering. Following this, clusters were manually grouped based on gene expression markers. Notably, cell state fractions were similar between surgical resection and EUS-FNB PDAC patients (Supplementary Fig. 5C).

### Cluster refinement

To exclude normal epithelial cells from the analysis, CopyKAT^23^ was used to identify cells with large numbers of copy number alterations (CNAs), and cells were further scored with a normal epithelial marker gene set from Cui Zhou et al.^20^ to identify putative normal epithelial cells (Supplementary Fig. 2A, B, Supplementary Table 4). Epithelial clusters with low numbers of CNAs and a high normal epithelial gene set score were excluded from downstream analysis. After 9k normal epithelial cells were removed, there were 76k malignant cells remaining. They were then labeled based on subtype markers previously described in the literature. We used 6 gene marker sets (Bailey et al., Moffitt et al., Chan-Seng-Yue et al., Raghavan et al., and Collisson et al.)^3,5,6,12,14^ for cluster assignment. Genes used for scoring each subtype are available in the supplemental materials (Supplementary Table 4). Ultimately, we partitioned the cells into two consensus subtypes: Basal-like and Classical.

The monocyte/DC cluster was further separated into the following five cell states based on marker expression and tumor-associated macrophage gene sets from Raghavan et al.^14^ Dendritic cells were separated into two groups: DC (FCER1A+) and pDC (BST2+). The following macrophage cell states were annotated based on enrichment for Raghavan et al. TAM gene sets: TAM–SPP1, TAM–C1QC, and TAM–FCN1 (Supplementary Table 4).

NK/T cells were separated based on the following marker genes: CD4 T cell (CD3G+, IL7R+), CD8 T cell (CD3G+, CD8A+), CD8 T cell exhausted (CD3G+, CD8A+, LAG3+, ITGAE+), T cell proliferating (CD3G+, TOP2A+), NK cell (GZMK+), and Treg (FOXP3+).

Fibroblasts were split into two groups of CAFs based on gene sets from Elyada et al.^17^ (Supplementary Fig. 3A, Supplementary Table 4).

### Bulk expression data acquisition

TCGA PDAC clinical and bulk RNA-seq expression data were downloaded from the NCI Genomic Data Commons (https://portal.gdc.cancer.gov/). Following removal of patients with <1 month survival and removal of neuroendocrine tumors, we were left with 136 tumors/patients (Supplementary Table 10). We then further restricted our analysis to samples reported in the study by Raphael et al. (*n* = 125 tumors/patients)^4^. Bailey et al.^5^ bulk RNA-seq and clinical data (*n* = 87 tumors/patients) were downloaded from the ICGC Data Portal (https://dcc.icgc.org/projects/PACA-AU). Kirby et al.^38^ bulk RNA-seq and clinical data (*n* = 45 tumors/patients) were downloaded from the GEO databank under GSE79670 (Supplementary Table 3).

TCGA COAD and HNSCC bulk RNA-seq datasets were downloaded from the NCI Genomic Data Commons (https://portal.gdc.cancer.gov/) (Supplementary Tables 13 and 14). The Mueller et al.^34^ murine microarray dataset was downloaded from the Gene Expression Omnibus (https://www.ncbi.nlm.nih.gov/geo/query/acc.cgi?acc=GSE107458).

### Ecotype scRNA-seq cell state discovery

Ecotype discovery was performed with a modified version of EcoTyper^28^. Four macro cell types in our labeled single-cell data could be broken down by cell state: Malignant (Basal-like and Classical), Fibroblast (myCAF and iCAF), Macrophage (TAM–C1QC, TAM–SPP1, TAM–FCN1, DC, pDC, DC/Macrophage–proliferating), NK/T cell (CD4 T cell, CD8 T cell, CD8 T cell exhausted, NK cell, Treg, T cell proliferating). The remaining cell types were treated as state-absent cell types (i.e., treated as a single cell state) and included endothelial cells, mast cells, erythrocytes, plasma cells, stellate cells, platelets, acinar cells, and B cells. Normal epithelial cells were excluded from the EcoTyper analysis.

The Ecotyper framework requires cell states to be present for all cell types, even those labeled as state-absent in our single-cell dataset. To remedy this, cell states were identified for the state-absent cell types by applying the EcoTyper scRNA-seq discovery framework to a subsampled scRNA-seq expression matrix (*N* = 17,033 cells) containing only state-absent cells. Discovered cell states for each state-absent cell type were then used in conjunction with the aforementioned manually identified single-cell states for downstream ecotype discovery.

The entire scRNA-seq expression dataset contained a total of ~190k cells, of which 50,435 originated from state-absent cell types. Of these 50,435 cells, 45,864 were labeled with discovered cell states, while the remaining 4571 cells were filtered out during the EcoTyper quality-control stage.

### Generation of the cell states coefficient matrix for EcoTyper

The published EcoTyper framework (Luca et al.^28^) applies non-negative matrix factorization (NMF) to infer cellular states from gene expression data.

Since we had already defined cellular states from our scRNA-seq dataset (as described above), we modified the EcoTyper framework to recover a basis matrix for downstream recovery when supplied with predefined cell state labels for specific cell types, thereby removing the need to conduct traditional NMF and discover de novo cell states. Specifically, let **G** represent a *g* × *n* cell type-specific scRNA-seq expression matrix **G** for cell type *i*, containing *g* genes along the rows and *n* samples (cells) along the columns. Given *s* cell states, for cell type *i*, let **H’** represent an *s* × *n* binary coefficient matrix with *s* cell states along the rows and *n* samples (cell) along the columns. For a sample (cell) *j*, if the prelabeled or assigned cell state is *q*, then **H’**(*q,j*) is set to **1**; otherwise, **H’**(*q,j*) is set to **O**. Thus, **H’** represents the membership of each sample (cell) to its respective prelabeled or assigned cell state and is fitted in a reference-based manner to recover **W’** for each cell type, which corresponds to a *g* × *s* basis matrix with *g* genes along the rows and *s* cell states along the columns and represents the average gene expression for each cell state. **W’** was then used in the traditional EcoTyper framework for cell state recovery in bulk RNA-seq expression data.

To perform ecotype discovery, a cell state abundance matrix was generated using the above cell state labels. Mapping was generated for cell types with predefined cell state labels; where each cell corresponds to a given cell state label. For cell types that were assigned cell states using the EcoTyper scRNA-seq discovery framework, the mapping was generated by the EcoTyper pipeline.

### Ecotype recovery

Human bulk RNA-seq expression data were TPM-normalized upon input to the EcoTyper framework. Murine microarray data were kept as raw values in non-logarithmic space. Ecotype recovery was then performed using the modified basis matrix **W**′ applied to each bulk gene expression cohort independently to recover cell states and ecotypes.

Recovery of carcinoma ecotypes (CEs) from Luca et al.^28^ was performed in line with the associated documentation (https://github.com/digitalcytometry/ecotyper) in *Tutorial 1: Recovery of Cell States and Ecotypes in User-Provided Bulk Data*.

### Kaplan–Meier analysis

Kaplan–Meier curves were generated with the Python lifelines package based on the most prevalent ecotype per patient tumor sample or the inferred developmental state of tumor cells or fibroblasts. For each survival analysis, log-rank p-values were computed with the lifelines logrank_test function using a one-versus-rest technique.

### Pseudo-bulk analysis

Pseudo-bulk mixtures were generated from the in-house EUS-FNB samples (*n* = 25) by summing read counts across all genes per sample. The total read counts matrix was TPM-normalized, and ecotypes were recovered by applying EcoTyper’s bulk recovery method as described in *Ecotype discovery and recovery*. Due to the modest sample size and to increase statistical power, ecotypes PE1 and PE6 were grouped and analyzed against PE5. Kaplan–Meier curves were generated with the Python lifelines package and statistical analysis was performed with the Gehan-Breslow-Wilcoxon test to calculate the *P*-value and Mantel–Haenszel method to calculate the hazard ratio using GraphPad Prism 9 (Supplementary Fig. 4B).

The same pseudo-bulk analytical procedure detailed above was used to classify ecotypes in the Lee et al.^35^ scRNA-seq COAD dataset.

### Development stemness analysis

CytoTRACE^24^ was independently applied to Malignant and Fibroblast scRNA-seq counts matrices to determine cellular developmental stemness. Counts matrices were CPM-normalized and run through CytoTRACE using default parameters. Differences in stemness between the Basal-like and Classical Malignant cell states and myCAF and iCAF Fibroblast cell states were determined using a two-sided Wilcoxon rank sum test (*p*-value « 0.05 for both cell types).

### Cell state-specific CytoTRACE gene distributions

Cell state-specific gene sets derived from EcoTyper were used to filter outputted CytoTRACE gene sets in order to retain gene expression profiles that were inferred to be specific to a given cell state. CytoTRACE values for these genes were then compared between cell states. High correlation values indicate genes that are associated with less differentiated, more stem-like cells. Significance values between distributions were determined using a two-sided Wilcoxon rank sum test.

### Bulk RNA-seq developmental score

Bulk RNA-seq developmental scores were calculated by taking the average gene expression of the top 20 CytoTRACE-correlated genes (those most associated with stemness) for fibroblast and malignant cells in the PDAC TCGA, Bailey et al., and Kirby et al. datasets^5,6,38^. The median developmental score for each cell type was used to partition samples into more stem-like versus less stem-like cell state groups. Significance between groups was calculated using the Wilcoxon rank sum test.

### Cox regression for overall survival

Univariate Cox proportional hazards regression was conducted for overall survival with respect to the bulk RNA-seq developmental score (described above) for malignant and fibroblast cell states in each dataset (Supplementary Table 11). Additional multivariate Cox proportional hazards regressions were conducted for overall survival in PDAC TCGA for PE5, along with clinical covariates including tumor stage, age, resection site, gender, race, treatment type, metastasis status, nodal status, and ethnicity (Fig. 3C, D, Supplementary Fig. 6A, Supplementary Table 12). Hazard ratios were calculated using the *exp(beta)* method, and covariate *p*-values were calculated using the Wald test.

### Gene set enrichment analysis (GSEA) and gene set scoring

Single-cell gene set scores were computed for various cell states by taking the mean expression of genes within the set. The gene sets used are available in Supplementary Table 4.

Pathway enrichment analysis for genes significantly associated with the myCAF cell state was done with the enrichrpy Python package (https://pypi.org/project/enrichrpy). Significant GO: Molecular Function pathways^45^ were selected based on enrichment of the top 30 differentially expressed genes in myCAFs (when compared to iCAFs). Top pathways were then rank-ordered by their −log10 FDR-corrected p-values.

### Reporting summary

Further information on research design is available in the Nature Research Reporting Summary linked to this article.

### Supplementary information

Supplementary Info

Supplemental Data

REPORTING SUMMARY

## Data Availability

Data for the scRNA-seq EUS-FNB cohort is available to download from GEO at accession number GSE242230. Data for the six scRNA-seq in-house surgical samples is available for download via dbGaP from the Human Tumor Atlas Network (HTAN) data portal under the Washington University Human Tumor Atlas Research Center (https://humantumoratlas.org/explore). Annotations and metadata for our single-cell dataset can be downloaded from Zenodo.

## Code availability

The code used in the modified EcoTyper framework is available in a public GitHub.
