# Supplementary material for: High-dimensional deconstruction of pancreatic cancer identifies tumor microenvironmental and developmental stemness features that predict survival
Source: NPJ Precis Oncol. 2023 Oct 19;7:105. doi: 10.1038/s41698-023-00455-z (PMC10587349; doi:10.1038/s41698-023-00455-z)
Supplement: Supplementary file 1 — Supplementary Info [file 41698_2023_455_MOESM1_ESM.pdf]

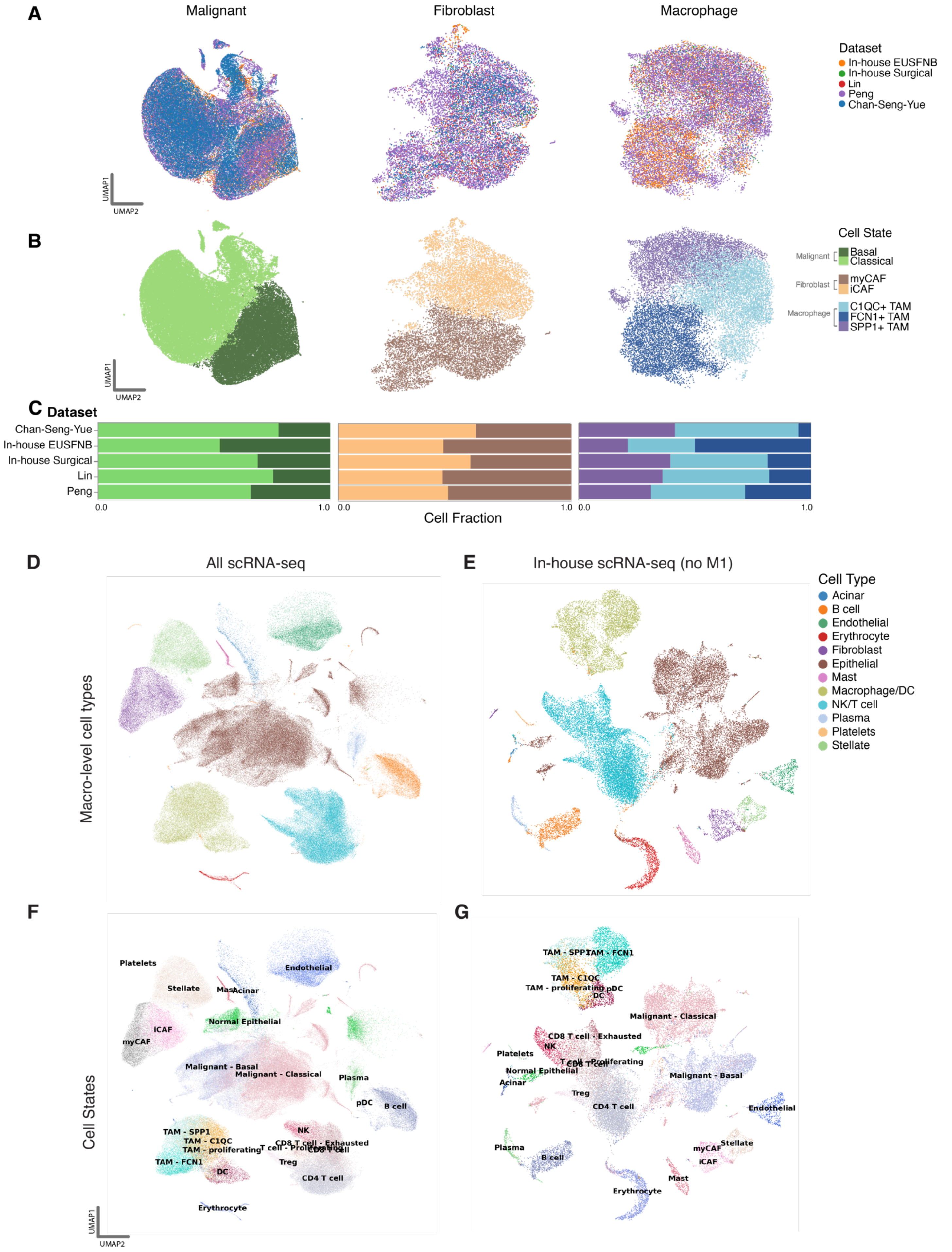
A
Malignant
Fibroblast
Macrophage
Dataset
In-house EUSFNB
In-house Surgical
Lin
Peng
Chan-Seng-Yue
UMAP1
UMAP2
B
Cell State
Malignant
Basal
Classical
Fibroblast
myCAF
iCAF
Macrophage
C1QC+ TAM
FCN1+ TAM
SPP1+ TAM
UMAP1
UMAP2
C
Dataset
Chan-Seng-Yue
In-house EUSFNB
In-house Surgical
Lin
Peng
0.0
1.0
0.0
1.0
0.0
1.0
Cell Fraction
D
All scRNA-seq
Macro-level cell types
E
In-house scRNA-seq (no M1)
Cell Type
Acinar
B cell
Endothelial
Erythrocyte
Fibroblast
Epithelial
Mast
Macrophage/DC
NK/T cell
Plasma
Platelets
Stellate
F
Cell States
Platelets
Stellate
Endothelial
Acinar
iCAF
myCAF
Normal Epithelial
Malignant - Basal
Malignant - Classical
Plasma
pDC
B cell
NK
TAM - SPP1
TAM - C1QC
TAM - proliferating
CD8 T cell - Exhausted
TAM - FCN1
DC
Treg
CD4 T cell
Erythrocyte
UMAP1
UMAP2
G
TAM - SPP1
TAM - FCN1
TAM - C1QC
TAM - proliferating
pDC
DC
Malignant - Classical
CD8 T cell - Exhausted
NK
Platelets
Normal Epithelial
Acinar
Malignant - Basal
Treg
CD4 T cell
Endothelial
Plasma
B cell
myCAF
Stellate
iCAF
Mast
Erythrocyte

Supplementary Figure 1. Single-cell integration. UMAP for malignant, CAF, and TAM populations colored by **A)** dataset of origin and **B**) assigned cell state. **C)** Malignant, CAF, and TAM cell fractions by dataset. UMAP decompositions for **D,F)** all scRNA-seq samples and **E,G)** in-house scRNA-seq with no metastatic patients at diagnosis colored by macro-level cell type and granular cell state annotations. The two sets of single cell data were normalized, integrated, and clustered separately.

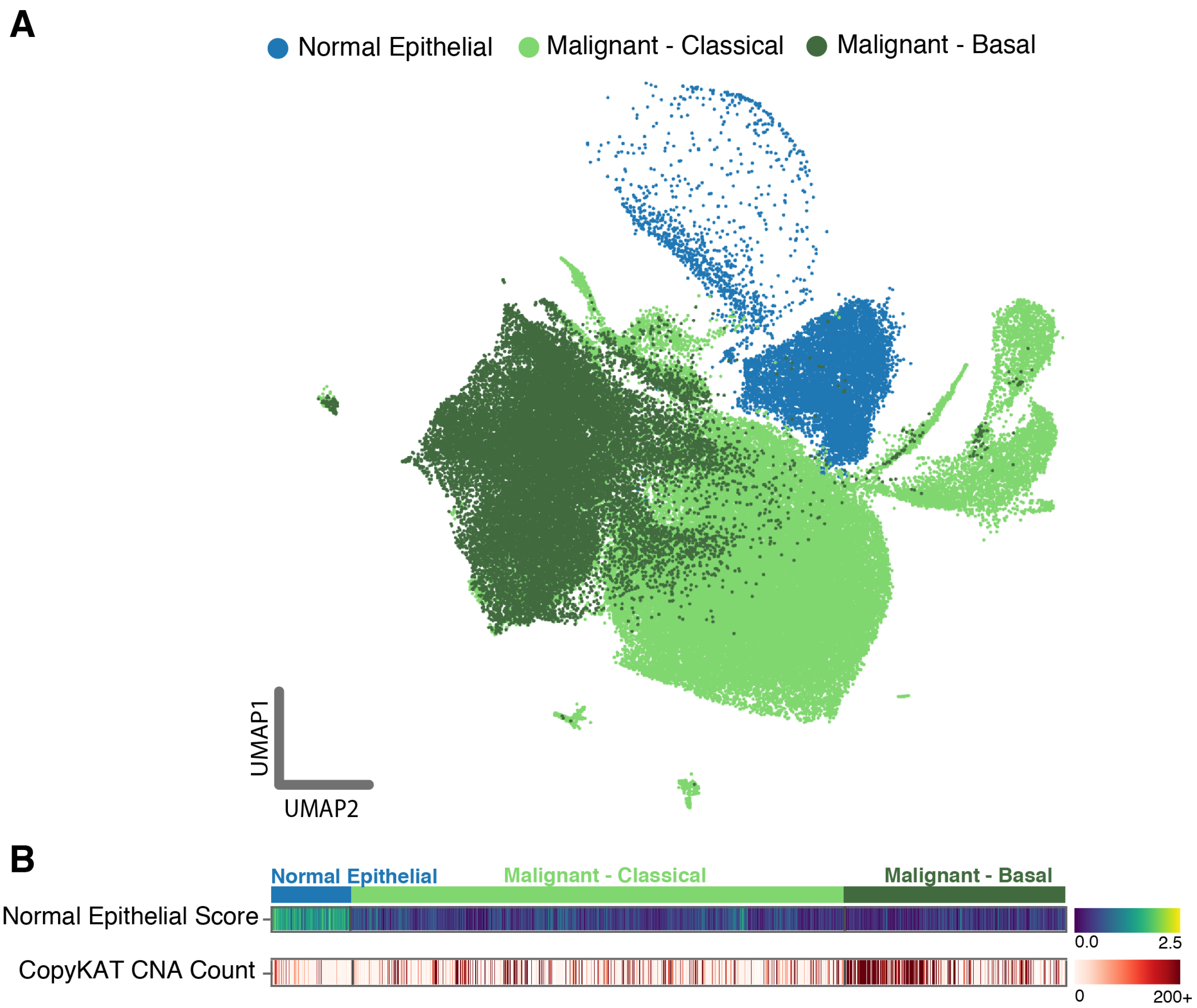

Supplementary Figure 2. Identification of different epithelial cell states. A) UMAP of all epithelial cells in scRNA-seq data, colored by epithelial cell state. **B)** Copy number alteration (CNA) count and normal epithelial gene set score for each cell analyzed by scRNA-seq, stratified by epithelial cell state.

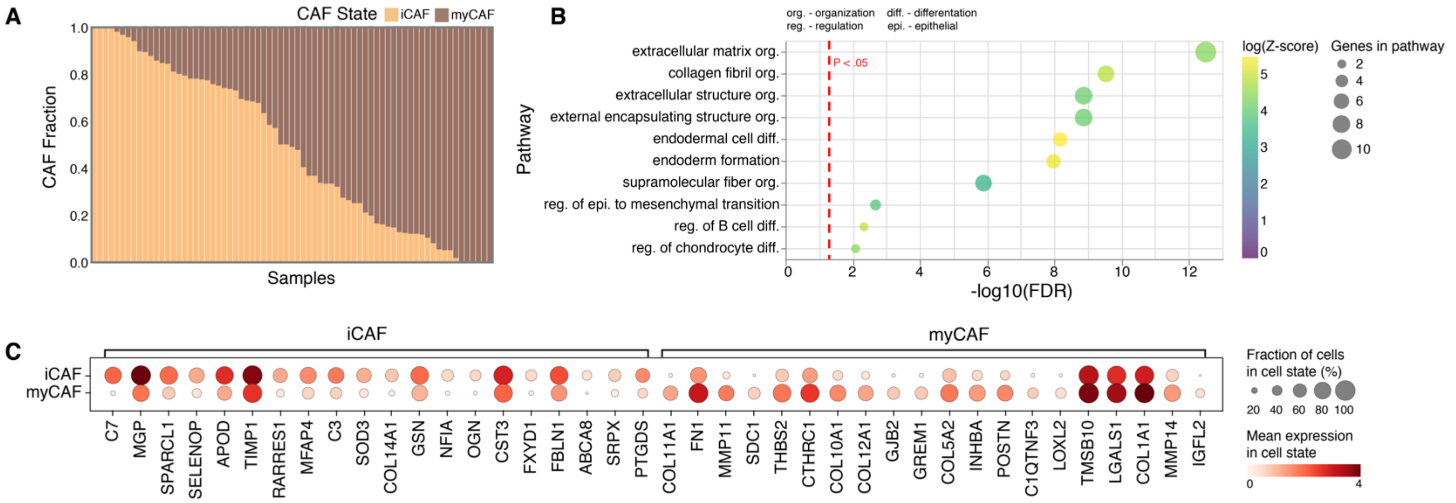

Supplementary Figure 3. CAF enrichment and pathway analysis. A) CAF cell state fraction for each scRNA-seq sample. **B)** myCAF pathway enrichment of Significant GO: Molecular Function pathways. **C)** Differentially expressed genes (DEGs) between iCAF and myCAF cell populations.

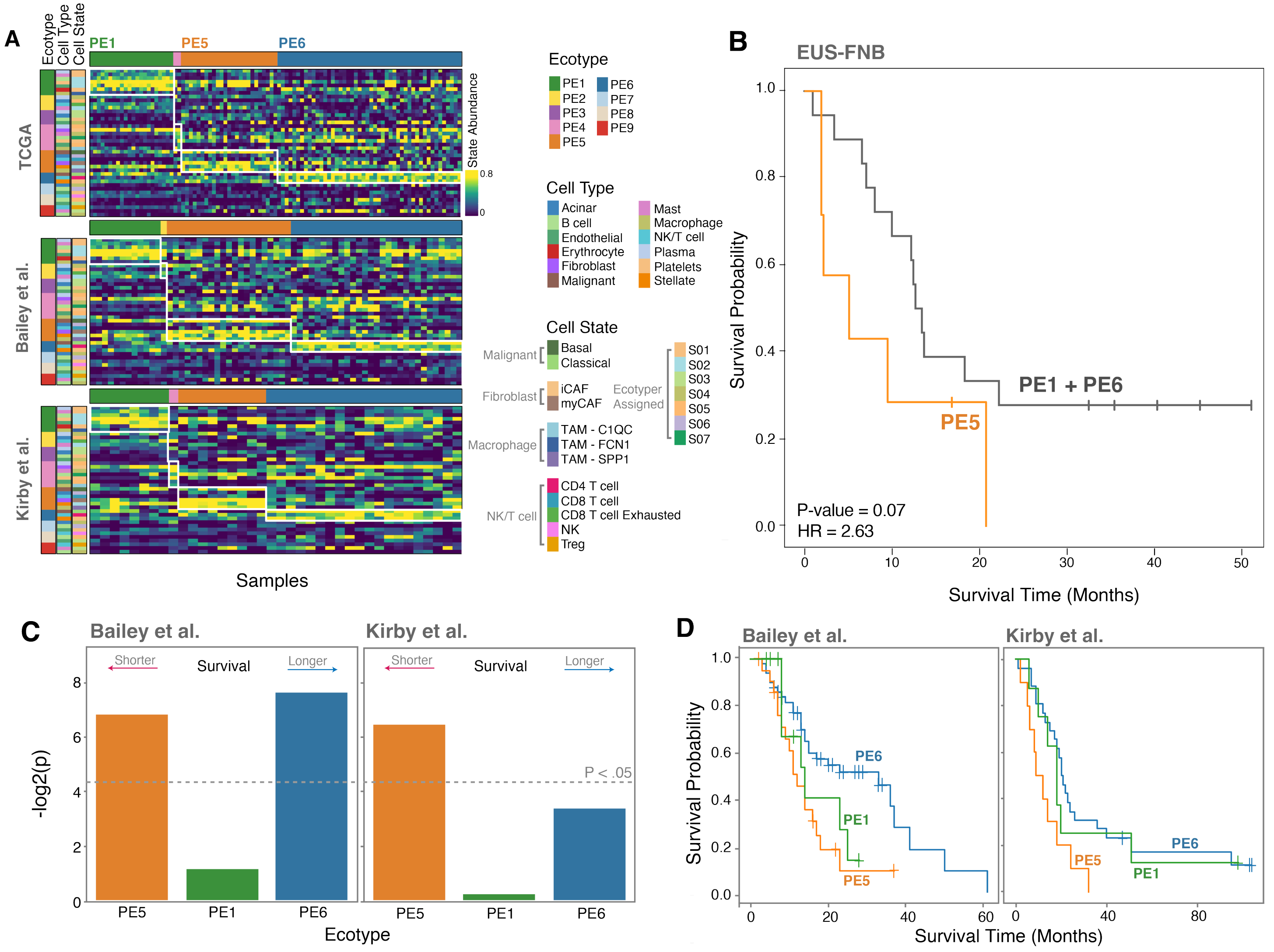

Supplementary Figure 4. Pancreatic ecotype associations with cell state abundance and survival across independent bulk datasets. A) Ecotype composition and cell state abundances for

TCGA (n = 125 tumors), Bailey et al. (n = 87 tumors), and Kirby et al. (n = 45 tumors) bulk RNA-seq datasets. **B)** Kaplan-Meier plots for EUS-FNB samples taken from the time-of-diagnosis (n = 25 patients) when pseudo-bulked and classified into ecotypes (p-value = 0.07, HR = 2.63). **C)** -log2(p-value) associations with survival for each major pancreatic ecotype in Bailey et al. and Kirby et al. **D)** Kaplan-Meier plots for PE1, PE5, and PE6 in Bailey et al. and Kirby et al.

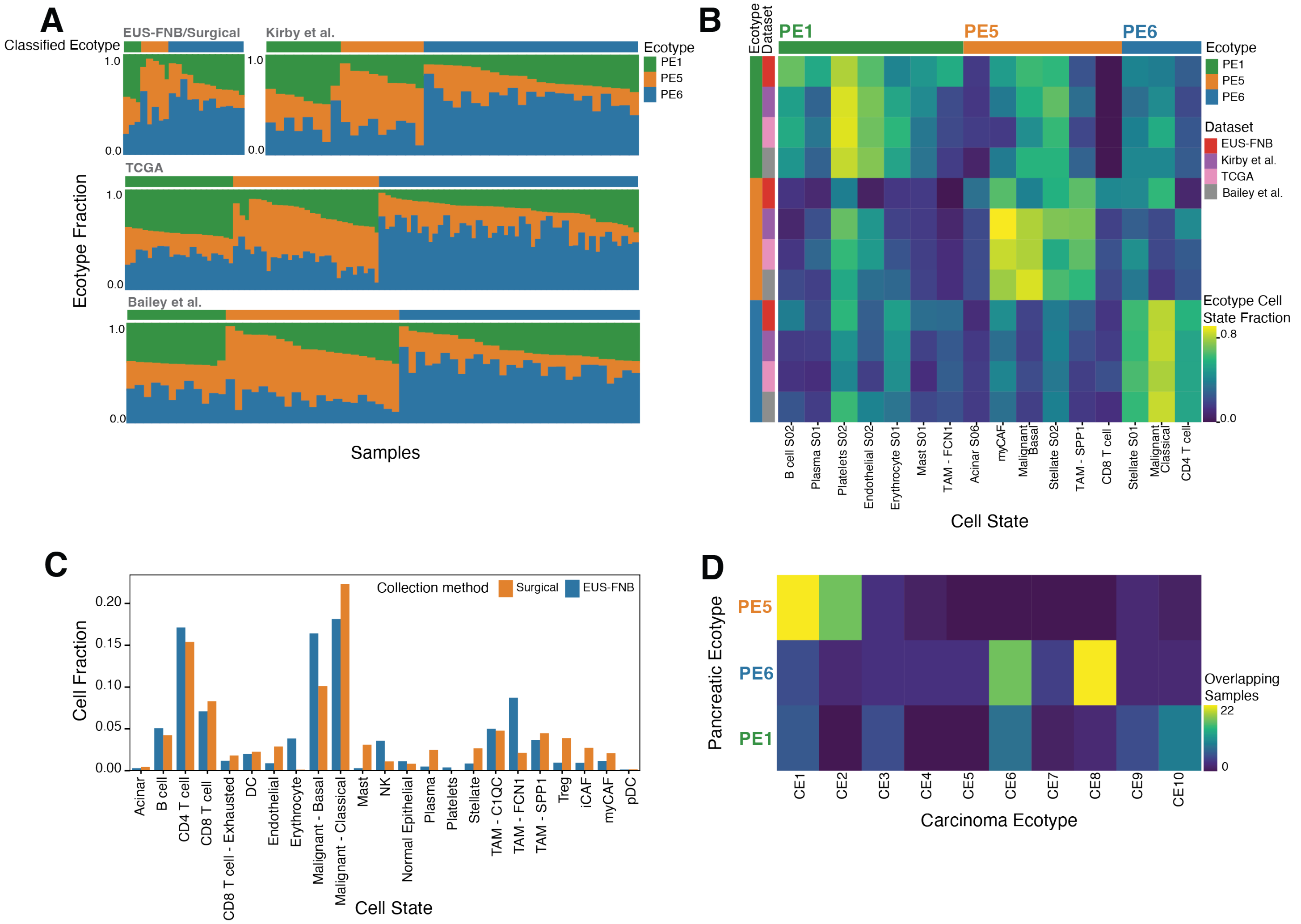

Supplementary Figure 5. Distributions across ecotypes in single cell and bulk samples and comparison to previously published ecotypes. A) Ecotype fractions for PE1, PE5, and PE6 ecotypes in in-house scRNA-seq and bulk RNA-seq cohorts. **B)** Ecotyper-inferred cell state abundances for cell states associated with PE1, PE5, and PE6 ecotypes across in-house scRNA-seq and bulk RNA-seq datasets. **C)** Comparison of cell state fractions in EUS-FNB versus surgical resection scRNA-seq in-house cohorts. **D)** Heatmap displaying overlap of ecotype classification between pancreatic ecotypes (PE1, PE5, PE6) and carcinoma ecotypes from Luca et al.

A

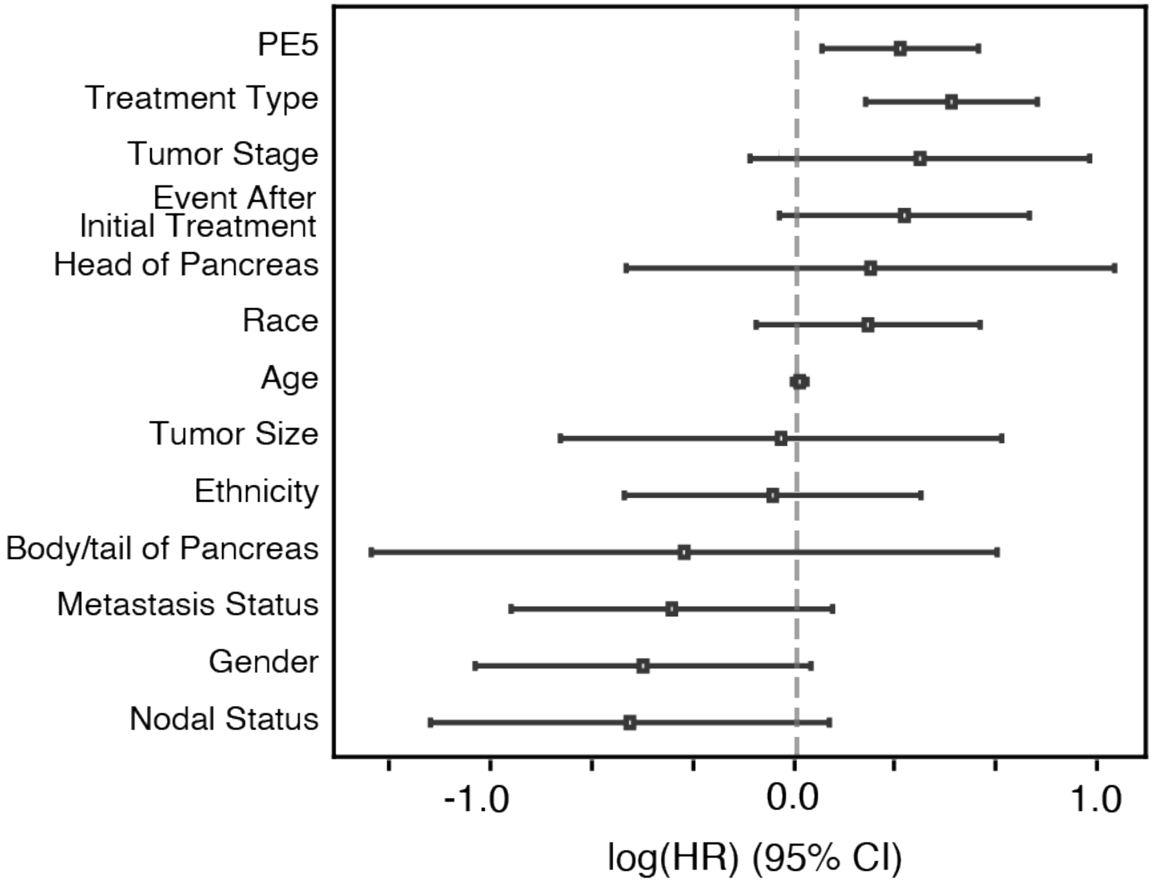

Supplementary Figure 6. Multivariate Cox regression analysis for overall survival including pancreatic ecotype status and clinical covariates. PE5 vs. PE1/PE6 ecotype classification in multivariate Cox proportional hazards regression analysis for overall survival along with clinical covariates in TCGA PDAC.

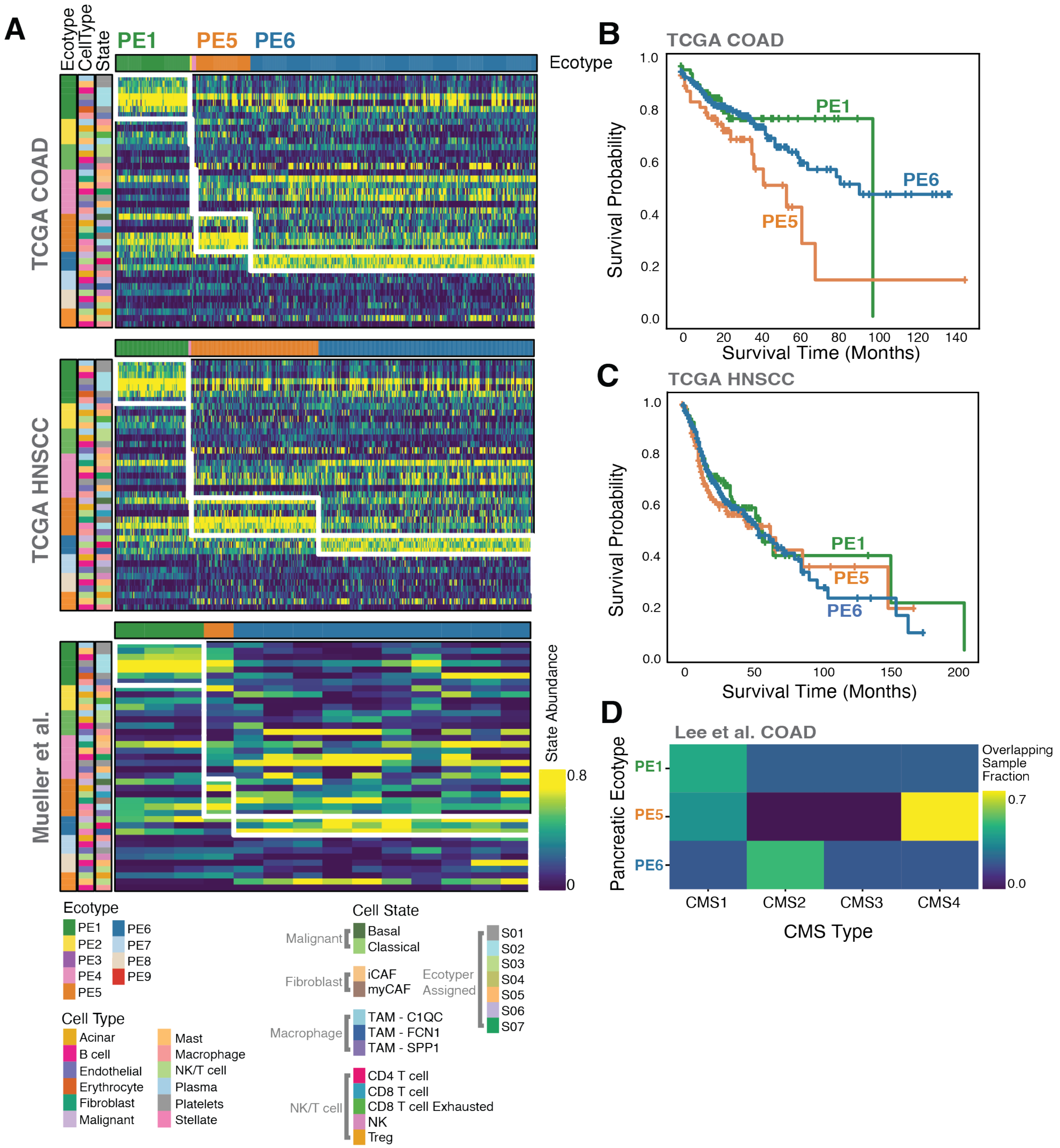

Supplementary Figure 7. Pancreatic ecotype classification across diseases and species. A) Pancreatic ecotype recovery of publicly available colorectal adenocarcinoma (COAD), head and neck squamous cell carcinoma (HNSCC), and PDAC murine samples from Mueller et al. Kaplan-Meier plots for **B)** TCGA COAD (p-value = 0.027) and **C)** TCGA HNSCC (p-value > .05) bulk RNA-seq samples when partitioned by pancreatic ecotypes. **D)** Overlap of pancreatic ecotype classifications and CMS subtypes in COAD scRNA-seq single-cell data from Lee et al.

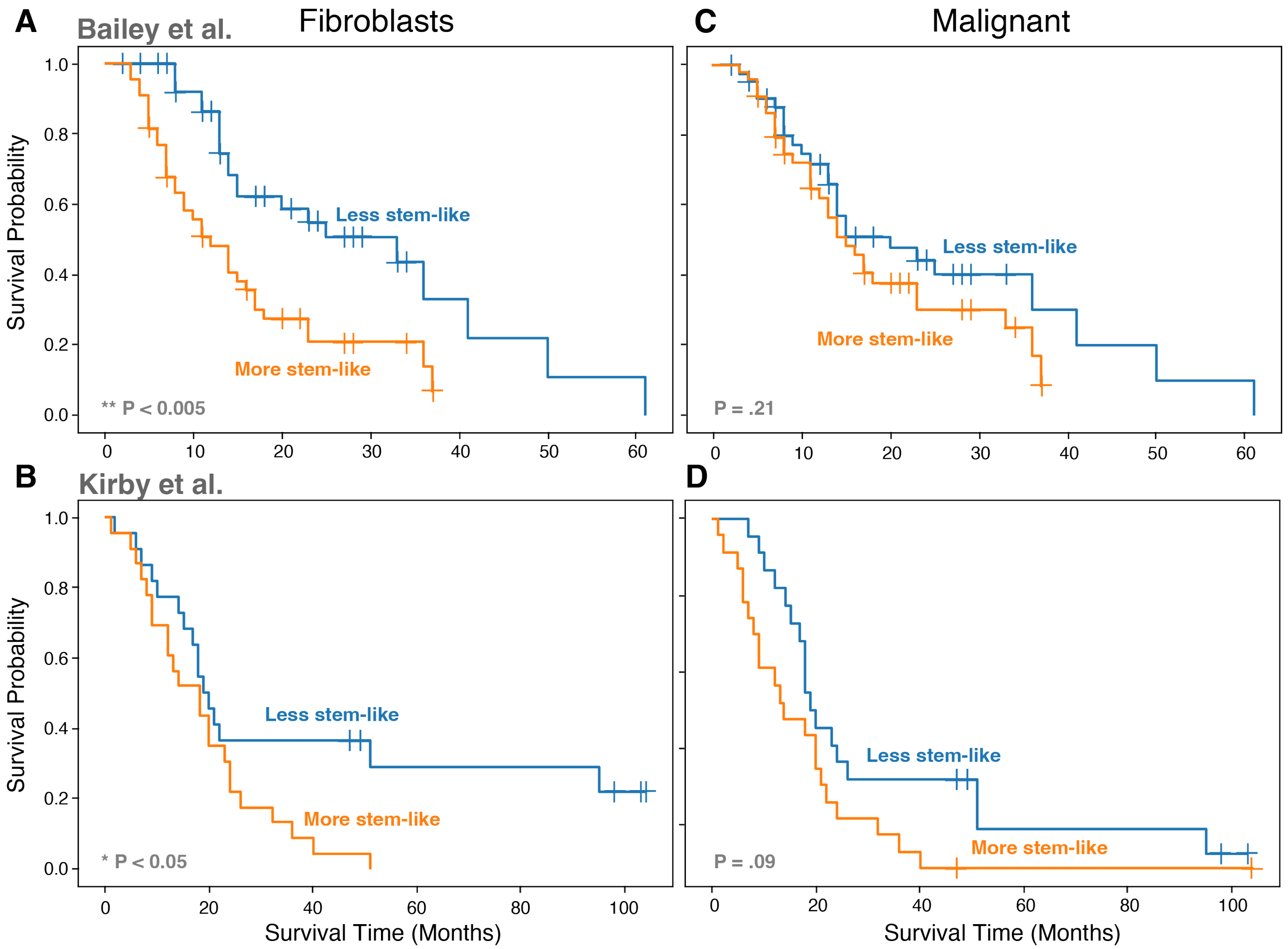

Supplementary Figure 8. Association of developmental stemness and survival across independent bulk datasets. A, B) Kaplan-Meier plots for bulk RNA-seq samples when partitioned into more versus less stem-like groups of fibroblasts in Bailey et al. (p-value = 0.0006) and Kirby et al. (p-value = 0.03). Groups were selected based on average Cytotrace correlation of cell type-specific genes. Mean score was used as a threshold to partition the two groups. **C, D)** Kaplan-Meier plots using the same methodology described above for malignant cells in Bailey et al. (p-value = 0.21) and Kirby et al. (p-value = 0.09).
