# Supplementary material for: High-dimensional deconstruction of pancreatic cancer identifies tumor microenvironmental and developmental stemness features that predict survival
Source: NPJ Precis Oncol. 2023 Oct 19;7:105. doi: 10.1038/s41698-023-00455-z (PMC10587349; doi:10.1038/s41698-023-00455-z)
Supplement: Supplementary file 3 — REPORTING SUMMARY [file 41698_2023_455_MOESM3_ESM.pdf]

nature portfolio

Corresponding author(s): Aadel A. Chaudhuri
Koushik K. Das

Last updated by author(s): Aug 30, 2023

Reporting Summary

Nature Portfolio wishes to improve the reproducibility of the work that we publish. This form provides structure for consistency and transparency in reporting. For further information on Nature Portfolio policies, see our Editorial Policies and the Editorial Policy Checklist.

Statistics

For all statistical analyses, confirm that the following items are present in the figure legend, table legend, main text, or Methods section.

n/a	Confirmed	
☐	☒	The exact sample size (*n*) for each experimental group/condition, given as a discrete number and unit of measurement
☐	☒	A statement on whether measurements were taken from distinct samples or whether the same sample was measured repeatedly
☐	☒	The statistical test(s) used AND whether they are one- or two-sided<br>*Only common tests should be described solely by name; describe more complex techniques in the Methods section.*
☐	☒	A description of all covariates tested
☐	☒	A description of any assumptions or corrections, such as tests of normality and adjustment for multiple comparisons
☐	☒	A full description of the statistical parameters including central tendency (e.g. means) or other basic estimates (e.g. regression coefficient) AND variation (e.g. standard deviation) or associated estimates of uncertainty (e.g. confidence intervals)
☐	☒	For null hypothesis testing, the test statistic (e.g. *F*, *t*, *r*) with confidence intervals, effect sizes, degrees of freedom and *P* value noted<br>*Give P values as exact values whenever suitable.*
☒	☐	For Bayesian analysis, information on the choice of priors and Markov chain Monte Carlo settings
☒	☐	For hierarchical and complex designs, identification of the appropriate level for tests and full reporting of outcomes
☒	☐	Estimates of effect sizes (e.g. Cohen's *d*, Pearson's *r*), indicating how they were calculated

Our web collection on statistics for biologists contains articles on many of the points above.

Software and code

Policy information about availability of computer code

Data collection	Spaceranger v2-3.2 was used to align scRNA-seq data.
Data analysis	Code used in the modified EcoTyper framework is available in a public Github repository at https://github.com/prathameshchati/Modified_EcoTyper_Code. Single-cell analysis was performed with scanpy v1.8.2 and Seurat v4. Survival analysis was performed with lifelines v0.27 and GraphPad PRISM v9.

For manuscripts utilizing custom algorithms or software that are central to the research but not yet described in published literature, software must be made available to editors and reviewers. We strongly encourage code deposition in a community repository (e.g. GitHub). See the Nature Portfolio guidelines for submitting code & software for further information.

Data

Policy information about availability of data

All manuscripts must include a data availability statement. This statement should provide the following information, where applicable:

- Accession codes, unique identifiers, or web links for publicly available datasets
- A description of any restrictions on data availability
- For clinical datasets or third party data, please ensure that the statement adheres to our policy

Data for the scRNA-seq EUS-FNB cohort is available to download from GEO at ascension GSE242230 (https://www.ncbi.nlm.nih.gov/geo/query/acc.cgi?acc=GSE242230). Data for the six scRNA-seq in-house surgical samples is available for download via dbGaP from the Human Tumor Atlas Network (HTAN) data

portal under the Washington University Human Tumor Atlas Research Center (https://humantumoratlas.org/explore). Annotations and metadata for our single cell dataset can be downloaded from Zenodo (https://zenodo.org/record/8301823).

Research involving human participants, their data, or biological material

Policy information about studies with human participants or human data. See also policy information about sex, gender (identity/presentation), and sexual orientation and race, ethnicity and racism.

Reporting on sex and gender	Gender was included as a clinical variable in our in-house cohorts and was self-reported. Gender was included as a covariate in survival analyses for bulk RNA-seq developmental score and PE5 vs. PE1/6 comparisons via multivariate cox proportional hazards modeling for TCGA PDAC in this study.
Reporting on race, ethnicity, or other socially relevant groupings	Race was self-reported as either white, black, or asian. Race and ethnicity (hispanic or other/NA) was used as a covariate in survival analyses for bulk RNA-seq developmental score and PE5 vs. PE1/6 comparisons via multivariate cox proportional hazards modeling for TCGA PDAC in this study.
Population characteristics	Age, treatment type, tumor stage, event after initial treatment, location of primary tumor, tumor size, metastasis status at diagnosis, and nodal status were included as covariates in multivariate cox proportional hazards modeling for TCGA PDAC. Population characteristics are summarized in Figure 1A and described in detail in the Methods.
Recruitment	All patients underwent written informed consent prior to tissue acquisition. Surgical resection (n=6) and EUS-FNB biopsy (n=25) specimens were included in this study. For EUS-FNB biopsy specimens, following written informed consent, endoscopic ultrasound was performed on patients with suspected solid pancreatic masses based on CT or MRI imaging. The diagnosis of pancreatic adenocarcinoma was confirmed by formal pathologic evaluation. After clinical diagnostic tissue acquisition was completed with 2-3 passes of a 22-gauge needle, an additional pass was obtained with a backfin “fine-needle biopsy” (FNB) needle.
Ethics oversight	Methods were performed in accordance with relevant guidelines and regulations and approved by the Institutional Review Board at the Washington University in St. Louis School of Medicine.

Note that full information on the approval of the study protocol must also be provided in the manuscript.

Field-specific reporting

Please select the one below that is the best fit for your research. If you are not sure, read the appropriate sections before making your selection.

☒ Life sciences ☐ Behavioural & social sciences ☐ Ecological, evolutionary & environmental sciences

For a reference copy of the document with all sections, see nature.com/documents/nr-reporting-summary-flat.pdf

Life sciences study design

All studies must disclose on these points even when the disclosure is negative.

Sample size	Pancreatic ductal adenocarcinoma (PDAC) primary tumor samples for our in-house single-cell discovery cohort were collected by endoscopic ultrasound-guided fine-needle biopsy (EUS-FNB) from 25 patients (31,215 cells) and surgical resections from 6 patients (11,353 cells). Our in-house single-cell cohort was integrated with 49 samples from three publicly available scRNA-seq datasets; the samples used were from Peng et al. (Genome Sequence Archive under project PRJCA001063), Lin et al. (GEO database at accession number GSE154778), and Chan-Seng-Yue et al. (EGA database under accession code EGAS00001002543). In total, 80 samples were obtained for the single-cell discovery cohort.<br><br>Publicly available PDAC bulk RNA-sequencing data were also utilized in this study; Data from 125 tumor samples were used from TCGA (NCI GDC), 87 tumor samples were used from Bailey et al. (ICGC Data Portal), and 45 tumor samples were used from Kirby et al. (GEO database under GSE79870). Publicly available clinical data associated with these three datasets were also downloaded and utilized. Seventeen additional PDAC murine microarray samples were also downloaded and used for analysis (GEO database under GSE107458).<br><br>Publicly available bulk and single-cell RNA-sequencing datasets were also downloaded and analyzed from colorectal adenocarcinoma (COAD) and head and neck squamous cell carcinoma (HNSCC) datasets. 518 HNSCC samples and 448 COAD samples were downloaded from TCGA (NCI GDC). Twenty-three COAD scRNA-seq samples were also used from the original EcoTyper paper (Luca et al., Cell, 2021) for analysis.
Data exclusions	No data were systematically excluded. All data included here met quality control thresholds as described in the Methods.
Replication	We performed ecotype discovery on an integrated dataset from 80 PDAC patients (31 in-house, 49 publicly available). Recovery of ecotypes was independently performed on 3 publicly available PDAC bulk RNA-sequencing cohorts and separately on a PDAC murine microarray cohort. Recovery of ecotypes was also independently performed on single-cell COAD data collected from the original EcoTyper manuscript (Luca et al., Cell, 2021) and two TCGA bulk RNA-sequencing datasets (COAD and HNSCC).
Randomization	Sample groups were defined based on sequencing methodology and biological/clinical phenotypes. No randomization was applied to our cohorts.
Blinding	Blinding was not utilized in this study. All samples were collected without prior knowledge of cell type and ecotype composition. Ecotype discovery was performed without the inclusion of survival data; survival analysis and clinical phenotype associations were independently

performed after ecotype discovery.

Reporting for specific materials, systems and methods

We require information from authors about some types of materials, experimental systems and methods used in many studies. Here, indicate whether each material, system or method listed is relevant to your study. If you are not sure if a list item applies to your research, read the appropriate section before selecting a response.

Materials & experimental systems

n/a	Involved in the study
☒	☐ Antibodies
☒	☐ Eukaryotic cell lines
☒	☐ Palaeontology and archaeology
☒	☐ Animals and other organisms
☒	☐ Clinical data
☒	☐ Dual use research of concern
☒	☐ Plants

Methods

n/a	Involved in the study
☒	☐ ChIP-seq
☒	☐ Flow cytometry
☒	☐ MRI-based neuroimaging
